# The Epigenome in Neurodevelopmental Disorders

**DOI:** 10.3389/fnins.2021.776809

**Published:** 2021-11-03

**Authors:** Julia Reichard, Geraldine Zimmer-Bensch

**Affiliations:** ^1^Functional Epigenetics in the Animal Model, Institute for Biology II, RWTH Aachen University, Aachen, Germany; ^2^Research Training Group 2416 MultiSenses-MultiScales, Institute for Biology II, RWTH Aachen University, Aachen, Germany

**Keywords:** epigenetics, corticogenesis, DNA methylation, histone modification, chromatin remodeling, non-coding RNAs, neuropsychiatry

## Abstract

Neurodevelopmental diseases (NDDs), such as autism spectrum disorders, epilepsy, and schizophrenia, are characterized by diverse facets of neurological and psychiatric symptoms, differing in etiology, onset and severity. Such symptoms include mental delay, cognitive and language impairments, or restrictions to adaptive and social behavior. Nevertheless, all have in common that critical milestones of brain development are disrupted, leading to functional deficits of the central nervous system and clinical manifestation in child- or adulthood. To approach how the different development-associated neuropathologies can occur and which risk factors or critical processes are involved in provoking higher susceptibility for such diseases, a detailed understanding of the mechanisms underlying proper brain formation is required. NDDs rely on deficits in neuronal identity, proportion or function, whereby a defective development of the cerebral cortex, the seat of higher cognitive functions, is implicated in numerous disorders. Such deficits can be provoked by genetic and environmental factors during corticogenesis. Thereby, epigenetic mechanisms can act as an interface between external stimuli and the genome, since they are known to be responsive to external stimuli also in cortical neurons. In line with that, DNA methylation, histone modifications/variants, ATP-dependent chromatin remodeling, as well as regulatory non-coding RNAs regulate diverse aspects of neuronal development, and alterations in epigenomic marks have been associated with NDDs of varying phenotypes. Here, we provide an overview of essential steps of mammalian corticogenesis, and discuss the role of epigenetic mechanisms assumed to contribute to pathophysiological aspects of NDDs, when being disrupted.

## Introduction

The mammalian brain is undoubtedly the most complex organ and challenges scientists in understanding its intricate morphology, connectivity and neuronal processing. Proper brain function critically relies on tightly orchestrated neurodevelopmental processes, which are by far not fully understood. In this context, various classes of neuropsychiatric and neurological disorders, including autism spectrum disorders (ASD), Tourette syndrome (TS), schizophrenia (SZ), bipolar disorder or different forms of epilepsy, are known to result from defective brain development (Niemi et al., 2018; Tărlungeanu and Novarino, 2018; López-Rivera et al., 2020; Vogel Ciernia et al., 2020; Arnett et al., 2021; Sidhaye and Knoblich, 2021). In general, such neurodevelopmental disorders (NDDs) affect 7-14% of all children in developed countries (Miller et al., 2016), manifest in that age and usually persist throughout life (Arnett et al., 2021). NDDs lead to severe physiological, psychological, cognitive and social impairments, and therefore drastically decrease life quality of affected individuals (Jonsson et al., 2017; Arnett et al., 2021). Due to their heterogeneity, NDDs cause multiple constellations of symptoms varying significantly across different diseases, creating treatment, prognosis and medical care difficult. Consequently, it is essential to determine underlying mechanisms and related risk factors causing neurodevelopmental defects. In addition to genetic risk factors, alterations in the epigenome have been proposed to be implicated in the disease etiology of several NDDs (Mastrototaro and Sessa, 2018). Due to the association with a broad range of NDDs, malformations of cortical development (MCDs) came to the fore over the last decades (Linde and Zimmer-Bensch, 2020; Subramanian et al., 2020). Although the exact incidence of affected individuals is unknown, it is suggested that MCDs cause nearly 75% of the reported cases of patients suffering from epileptic seizures (Leventer et al., 1999), and 40% of cases of intractable or medication-resistant childhood epilepsies (Barkovich et al., 2012; Guerrini and Dobyns, 2014). To dissect underlying pathophysiological processes that lead to anatomic malformations and functional defects seen in NDDs and MCDs, it is important to understand how the cortex develops, and how genetic and epigenetic risk factors cause such dysregulation of neurodevelopment. The following passages provide an overview of the most important milestones of cortical development and its spatiotemporal regulation in humans and mice. Moreover, the present review highlights defects occurring during corticogenesis, which are discussed to cause NDDs, as well as implications of the epigenome in the regulation of proper and defective development of the neocortex.

## Principles of Neocortical Development

**FIGURE 1:**
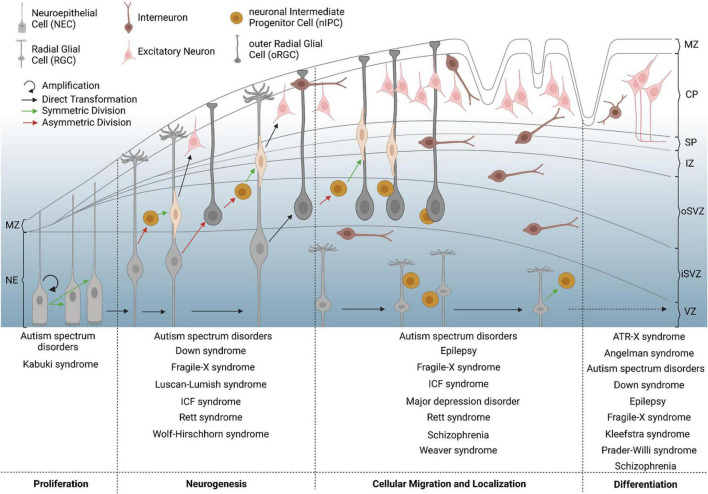
Critical milestones of human corticogenesis and associated NDDs. The human cerebral cortex begins to form by symmetric division of neuroepithelial cells (NECs, bright gray) in the first trimester, which elongate in shape to convert to radial glial cells (RGCs, dark gray). RGCs increase in number, asymmetrically divide and convert to outer radial glial cells (oRGCs) also known as basal RGCs, or give rise to neuronal intermediate progenitor cells (nIPCs), again by asymmetric division. The latter further divide symmetrically to give rise to young excitatory principal neurons (pink), which migrate from the subventricular zone (SVZ) toward the forming cortical plate (CP). Inhibitory interneurons (brown) invade the developing neocortex along the marginal zone (MZ) or the subplate (SP) and SVZ, before they switch to radial migration to enter the cortical plate. Increased progenitor and neuronal numbers as well as rapidly expanding neuronal networks contribute to physical stress, forming the main gyri at the end of the second trimester. At later stages of corticogenesis, intercellular connections begin to form, for which morphological differentiation and defined setting of neuronal proportions are necessary. Failures of corticogenesis are suggested to contribute to various NDDs with respect to the given time point and affected process, which is depicted on the bottom for different examples of diseases. CP = cortical plate; iSVZ = inner subventricular zone; IZ = intermediate zone; MZ = marginal zone; NE = neuroepithelium; oSVZ = outer subventricular zone; SP = subplate; VZ = ventricular zone.

The proper formation of the mammalian neocortex is the result of closely controlled processes including progenitor proliferation and differentiation, cellular migration, morphological maturation and the establishment of synaptic contacts, as well as programmed cell death, resulting in functional circuits of billions of morphologically and functionally distinct neurons (Figure 1; Hu et al., 2014; Kalebic et al., 2017; Lim et al., 2018; Mukhtar and Taylor, 2018; Subramanian et al., 2020). Neuronal circuits of the neocortex are composed of two major types of cortical neurons: excitatory principal neurons expressing glutamate, and the inhibitory γ-aminobutyric acid (GABA)-expressing local interneurons. Although inhibitory interneurons constitute the minority of the entire neuronal population within the cortex with 25-30% in humans and 15-20% in mice, they critically maintain cortical functionality in both species, establishing a balanced equilibrium of cortical excitation and inhibition (Jones, 2009; Huang and Paul, 2018; Sultan and Shi, 2018).

The proportionally larger population of excitatory principal neurons (70-85% of the neuronal cells in the cortex), are classified based on the respective laminar location, axonal projections, dendritic morphology, as well as the biochemical marker expression (Bayer and Altman, 1991; Lodato et al., 2011a; Greig et al., 2013). Additionally, recent studies suggest that neuronal cell types differ between cortical areas in regard to their transcriptomic profiles (Saunders et al., 2018; Tasic et al., 2018; Cadwell et al., 2020). One major group of excitatory neurons within the mammalian cortex are the spiny stellate cells located in layer IV, which are characterized by several dendrites of similar lengths and which mainly project locally to areas near their soma (Shepherd, 2004; Costa and Müller, 2015). Contrarily, pyramidal cells have long projecting axons and apical dendrites for which they are also called projection neurons (Costa and Müller, 2015). This subgroup constitutes the majority of cortical excitatory neurons and localizes in all layers of the mammalian neocortex except for layer I (Costa and Müller, 2015). The excitatory principal neurons arise from the proliferation zone of the dorsal telencephalon and migrate along the radial-glial scaffold composed of basal processes of radial glial cells (RGCs) toward the pial surface, forming the cortical plate as a transient developmental structure (Figure 1; Costa and Müller, 2015; Mukhtar and Taylor, 2018; Sultan and Shi, 2018; Subramanian et al., 2020). Here, they build the cortical layers in an inside-out fashion, with early-born neurons giving rise to the deeper cortical layers and late-born neurons constituting the superficial layers (Angevine and Sidman, 1961; Fairén et al., 1986; López-Bendito et al., 2004; Greig et al., 2013).

The second major class of cortical neurons, the inhibitory GABA-expressing interneurons, represent a highly diverse group within the cortex of rodents, humans, non-human primates and mustelids (Guo and Anton, 2014; Costa and Müller, 2015; Lim et al., 2018; Sultan and Shi, 2018; Zimmer-Bensch, 2018). They are distinguished according to morphological, electrophysiological, and biochemical properties (Nery et al., 2002; Bystron et al., 2008; Fishell, 2008; Gelman et al., 2009; Miyoshi et al., 2015; Hatanaka et al., 2016; Bandler et al., 2017; Wamsley and Fishell, 2017; Lim et al., 2018; Symmank et al., 2019). In contrast to excitatory neurons, the distinct inhibitory cortical interneuron subtypes originate in particular domains of the basal telencephalon (Lim et al., 2018; Mukhtar and Taylor, 2018; Subramanian et al., 2020). The medial ganglionic eminence (MGE) gives rise to parvalbumin (PV)-positive basket and chandelier cells, and somatostatin (SST)-expressing Martinotti and multipolar interneurons. The pre-optic area (POA) generates neuropeptide Y (NPY)-, reelin-, SST-, and COUP-TF-interacting protein-2 (CTIP2)-expressing interneurons. Moreover, the caudal ganglionic eminence (CGE) contributes to reelin-expressing interneurons, vasointestinal-peptide- (VIP)/calretinin-positive bipolar cells, as well as VIP-/cholecystokinin-expressing basket cells (Gelman et al., 2011; Zimmer-Bensch, 2019b). Thereby, most interneurons are born in the MGE and the dorsal part of the CGE (dCGE), with smaller subsets being generated in the subpallial septum and the POA (Gelman et al., 2009; Marín et al., 2010; Faux et al., 2012; Lim et al., 2018; Sultan and Shi, 2018; Zimmer-Bensch, 2018). In humans and monkeys, smaller populations of GABAergic interneurons appear to additionally originate from dorsal parts of the telencephalon, however with a temporal delay compared to ventrally located eminences, assuming an evolutionary strategy of primate corticogenesis (Petanjek et al., 2009; Krienen et al., 2020).

Deciphering how the neocortex develops in primates and rodents has helped to shed light on important aspects of corticogenesis that are known to be disturbed in NDD-associated contexts (Bayer and Altman, 1991; Palmini et al., 1991; Leventer et al., 1999; Gleeson, 2001; Barkovich et al., 2012; Sun and Hevner, 2014; Lamsal and Zwicker, 2017; Hamm et al., 2020; Linde and Zimmer-Bensch, 2020; Subramanian et al., 2020). In humans, corticogenesis starts in the first trimester in the dorsal telencephalon and leads to rapid expansion in size, connectivity and structural organization (García-Moreno et al., 2012; Sun and Hevner, 2014; Penisson et al., 2019; Subramanian et al., 2020). Polarized neuroepithelial cells (NECs), which line the lumen of the neural tube, undergo symmetric divisions, through which the progenitor pool is increased, and finally elongate in cell shape becoming RGCs (Figure 1; Kriegstein and Alvarez-Buylla, 2009; García-Moreno et al., 2012; Sun and Hevner, 2014; Penisson et al., 2019). These cells either undergo symmetric proliferative division to increase their number, or asymmetrically divide to generate neurons, which is called “direct neurogenesis” (Kriegstein and Alvarez-Buylla, 2009; García-Moreno et al., 2012; Sun and Hevner, 2014; Agirman et al., 2017; Borrell, 2019; Penisson et al., 2019). In addition, intermediate progenitor cells (IPCs) are produced by asymmetric division, which then generate neurons by “indirect neurogenesis” through symmetric divisions in the subventricular zone (SVZ), where they re-locate after losing their apical and basal contacts (Kriegstein and Alvarez-Buylla, 2009; Agirman et al., 2017; Borrell, 2019; Penisson et al., 2019). Further insights in generation and specification of neuronal progenitor cells as well as comprehensive aspects of neurogenesis have been widely described in several publications over the last years (Taverna et al., 2014; Molnár et al., 2019; Kalebic and Huttner, 2020; Llorca and Marín, 2021). While it was initially suggested that indirect neurogenesis by IPCs mainly gives rise to upper-layer neurons (Tarabykin et al., 2001; Nieto et al., 2004; Roy et al., 2004), recent studies by Cárdenas et al. (2018) and Vitali et al. (2018) propose indirect neurogenesis as major mode of producing neurons fated for the deep as well as superficial cortical layers during murine corticogenesis. The developing human cortex displays an inner SVZ (iSVZ) and outer SVZ (oSVZ), which host basal intermediate progenitor cells (bIPCs) and basal RGCs (bRGCs) (Figure 1; Sun and Hevner, 2014). While less prominent, bRGCs are also seen in the SVZ of the murine cortex (García-Moreno et al., 2012; Sun and Hevner, 2014). As bRGCs are highly prominent in humans and mammals with a high rate of gyrification, such as ferrets, while being rather few in number in lissencephalic species such as the mouse (Penisson et al., 2019; Subramanian et al., 2020), bRGCs have been associated with the generation of cerebral gyrification and are of critical importance for cortical folding. In addition to that, gyrification is hypothesized to be determined by neuro- and gliogenesis, progenitor cell density, cellular migration, and the formation of cortical connections with respect to individual regions of gyri and sulci (Penisson et al., 2019; Wang et al., 2020). In line with the aforementioned terminology, the RGCs and IPCs present in the human and murine ventricular zone (VZ) are named apical IPCs (aIPCs) and apical radial glial cells (aRGCs) (García-Moreno et al., 2012; Sun and Hevner, 2014).

Upon becoming post-mitotic, young neurons display characteristic migratory behaviors, which differ between excitatory principal neurons and inhibitory interneurons (Figure 1; Agirman et al., 2017; Lim et al., 2018; Mukhtar and Taylor, 2018; Zimmer-Bensch, 2018; Weng et al., 2019; Subramanian et al., 2020). Post-mitotic excitatory neurons, generated in the cortical proliferative zones, migrate out of the VZ and SVZ along the scaffold of radial glial cells, through the inner and outer fiber layer (iFL and oFL) in humans and through the intermediate zone (IMZ) in rodents (Zimmer-Bensch, 2019a). Subsequently, they invade the cortical plate and localize within respective laminar positions, generating apical dendrites and small axons (Zimmer-Bensch, 2019a). These axons and newly formed intercellular connections become myelinated at perinatal stages by oligodendrocytes in humans (Kostović and Judaš, 2015; Subramanian et al., 2020). In rodents, the majority of cortical cells gets myelinated at P10-35 (Kostović and Judaš, 2015). Initially, it was assumed that myelination occurs homogenously across all cortical layers. However, previous observations by Tomassy et al. (2014) indicate a gradual myelinization of projection neurons located in different layers, potentially contributing to functional diversity of excitatory neuron populations.

Compared to projection neurons, cortical interneurons perform glial cell-independent long-range migration through the basal telencephalon toward the cortex, following defined and origin-specific routes, guided by various spatially and temporally expressed chemoattractive and -repellent signaling molecules (Marín et al., 2003, 2010; Zimmer et al., 2007, 2010, 2011; Petanjek et al., 2009; Rudolph et al., 2010, 2014; Friocourt and Parnavelas, 2011; Faux et al., 2012; Guo and Anton, 2014; Symmank et al., 2019). In the mouse embryo, migration starts around E11.5 from the MGE. At mid-embryonic stage E12.5-E14.5, migration of MGE and POA-derived cells along deep and superficial migratory streams has been documented (Marín and Rubenstein, 2001; Symmank et al., 2019). Finally, interneuron migration from the CGE starts around E13.5 (Nery et al., 2002). At later embryonic stages (E14.5-16.5), migrating interneurons tangentially invade the cortex along two major streams, through the IZ, SVZ and subplate, as well as through the marginal zone (MZ) (Figure 1; Tanaka and Nakajima, 2012; Guo and Anton, 2014). Of note, excitatory projection neurons already differentiate and begin to form layers in the developing cortical plate at this embryonic stage (López-Bendito et al., 2008). After tangential spreading over the cortical areas, interneurons switch from a tangential to a radial mode of migration to invade the cortical layers in part with the help of glial processes (Figure 1; Hatanaka et al., 2016). However, also glial cell-independent radial migration of cortical interneurons has been documented (Yokota et al., 2007). Moreover, intracortical migration of GABAergic interneurons has been shown to occur in a multidirectional and random fashion (Polleux et al., 2002; Rymar and Sadikot, 2007; Tanaka et al., 2009; Faux et al., 2012; Marín, 2013; Bartolini et al., 2017).

Correct numbers of excitatory and inhibitory neurons guarantee proper cortical function. Thereby it seems to be an evolutionarily conserved strategy to overproduce developing cortical neuron populations with subsequent fine-tuning of neuronal numbers by controlled cell death (Wong and Marín, 2019). Additionally, early-born neurons, such as Cajal-Retzius cells and subplate neurons are also reduced by apoptotic events to adjust final cellular numbers (Wong and Marín, 2019). In case of GABAergic interneurons about half of their embryonic population is diminished within early postnatal days of mouse pups (Yamaguchi and Miura, 2015), whereas for inadequate or dysregulated projections from pyramidal cells these connections are also eliminated at this stage (Raff, 1992). Besides post-migratory regulation of cellular survival, there is also evidence for survival regulation during cellular migration involving the transcription factor LHX1 (Symmank et al., 2019).

In addition to the influence of the local environment and extracellular stimuli, distinct aspects of neuronal fate determination, migration and morphological maturation rely on intrinsic transcriptional networks, as revealed by diverse fate mapping and clonal analyses studies (Mayer et al., 2015, 2016; Pensold and Zimmer, 2018; Zimmer-Bensch, 2018; Guzelsoy et al., 2019; Paolino et al., 2020), which will be discussed as follows.

### Transcriptional Networks Regulating Neuronal Identity Transition, Migration, and Maturation

Transcriptional networks were shown to regulate cortical neurogenesis, neuronal identity, cellular migration, cortical positioning, and the formation of neuronal connections (Table 1; Konopka et al., 2012; Sandberg et al., 2018; Guzelsoy et al., 2019; Paolino et al., 2020), whereby numerous questions still remain open or debated. In matters of the determination of cortical interneuron identity, two different assumptions are discussed in the literature (Fishell, 2008; Wamsley and Fishell, 2017). On the one hand, the *progenitor specification hypothesis* suggests that interneuron cell fate is determined already at progenitor level relying on intrinsic transcriptional programs (Fishell, 2008; Quattrocolo et al., 2017). For example, the development of MGE-derived interneurons is characterized by the progressive expression of different transcription factors, starting with *Nkx2.1* within progenitor cells (Polleux et al., 2002; López-Bendito et al., 2004), followed by transient expression of *Lhx6*, *Sox6*, and *Sip1* during migration to the cortex, and *Satb1* as soon as MGE-cells invade the cortex (Faux et al., 2012; Wamsley and Fishell, 2017). Hence, the NKX-class homeobox transcription factor 2.1 (NKX2.1) represents the top of the transcriptional cascade, determining MGE fate and migration (Sandberg et al., 2016). In turn, the transcription factor LIM homeobox 6 (LHX6) primes MGE-derived interneuron development through transcriptional regulation of the migration-associated genes *Cxcr7, Satb1* and *Arx*, or drives expression of *Sox6*, which is essential for interneuron migration and laminar positioning (Faux et al., 2012; Wamsley and Fishell, 2017; Zimmer-Bensch, 2018). Early specification of neuronal fate underlining the progenitor specification hypothesis is also described for interneurons deriving from the POA. Here, the transcription factors NKX6.2 and DBX1 are reported to be expressed in the dorsal versus ventral neuroepithelium of the POA, driving the specification of POA-derived interneuron progenitors (Flames et al., 2007). However, using clonal strategies in embryonic MGE-derived progenitors, recent studies indicated that information at progenitor level seems sufficient to determine the subsequent allocation to particular cortical regions or subtype identity (Mayer et al., 2015, 2016).

**TABLE 1:** Transcriptional regulation in mammalian corticogenesis.

Developmental process	Transcription factor	Regulated targets
Proliferation	CTNB1 Faux et al., 2012 transcriptional co-activation WNT/β-catenin Ahmed et al., 2009 HES1/5 Ahmed et al., 2009 GLI1-3 Ahmed et al., 2009 SOX1-3 Ahmed et al., 2009 REST Ahmed et al., 2009 SOX9 Fabra-Beser et al., 2021 YAP1 Fabra-Beser et al., 2021 PAX6 Sansom et al., 2009	Tcf/Lef, *Fgf2* Ahmed et al., 2009 *Sox2*, *Hes1*, *Hes5*, *Notch1*, *Cd133*, *Bmi-1* Ahmed et al., 2009 *Sox9* Fabra-Beser et al., 2021 *Neurog2*, *Ascl1*, *Hes1* Sansom et al., 2009
Differentiation	INSM1 Hevner, 2019 NEUROD2* Guzelsoy et al., 2019 FEZF2* Lai et al., 2008 CTIP2* Lai et al., 2008 TBR1/2* Hevner, 2019 CUX1/CUX2**°** Faux et al., 2012 DLX1,2,5,6° Faux et al., 2012 LHX6,8° Faux et al., 2012 MASH1° Faux et al., 2012 PAX6° Zimmer-Bensch, 2018 SOX2° Zimmer-Bensch, 2018 CBF1 Ahmed et al., 2009	*Neurod1* Hevner, 2019 *Pax6, Insm,1 Robo2, Fog2, Zfp423* Hevner, 2019 *Zeb2* McKinsey et al., 2013 *Sox2, Nanog, Hes1* Zimmer-Bensch, 2018 *Ascl1, Dlx1* Zimmer-Bensch, 2018
Specific interneuron lineage fate MGE-derived CGE-derived POA-derived	ZEB2 Zimmer-Bensch, 2018 NKX2.1 Sussel et al., 1999; Du et al., 2008; Faux et al., 2012 LHX6 Liodis et al., 2007; Faux et al., 2012; Neves et al., 2013 NKX6.2 SOX6 Batista-Brito et al., 2009; Faux et al., 2012; Wamsley and Fishell, 2017 SIP1 van den Berghe et al., 2013; Wamsley and Fishell, 2017 SATB1 Close et al., 2012; Denaxa et al., 2012; Wamsley and Fishell, 2017 Coup-TFI/II Zimmer-Bensch, 2018 Coup-TFI/II Faux et al., 2012 PROX1 Miyoshi et al., 2015; Zimmer-Bensch, 2018 SP8 Zimmer-Bensch, 2018 NR2F2 Zimmer-Bensch, 2018 NKX5.1/HMX3 Gelman et al., 2009; Gelman and Marín, 2010 LHX1 Symmank et al., 2019 NKX6.2 Flames et al., 2007; Gelman et al., 2009; Gelman and Marín, 2010	*Nkx2.1, cMaf, Cxcr7* Zimmer-Bensch, 2018 *Lhx6* Du et al., 2008 *Arx, Cxcr7, Sox6, Satb1* Zimmer-Bensch, 2018 *Sox6* Zimmer-Bensch, 2018
	DBX1 Flames et al., 2007; Gelman et al., 2009; Gelman and Marín, 2010	
Interneurons tangential migration	ZEB2 McKinsey et al., 2013; Zimmer-Bensch, 2018 Coup-TFI/II Faux et al., 2012; Zimmer-Bensch, 2018 DCX Faux et al., 2012 DLX1,2 Faux et al., 2012 LHX6 Faux et al., 2012; Zimmer-Bensch, 2018	*Unc5b* McKinsey et al., 2013; Zimmer-Bensch, 2018 *Arx, Cxcr7* Zimmer-Bensch, 2018
Interneuron guidance through ventral telencephalon	LHX1 Symmank et al., 2019	*Epha4, efnB3* Symmank et al., 2019
Radial migration	SOX5* Lai et al., 2008 NGN2* Hand and Polleux, 2011 MEF2C* Hand and Polleux, 2011 NEUROD1* Hand and Polleux, 2011 NEUROD2* Hand and Polleux, 2011 SOX6° Faux et al., 2012 KIR2.1° García et al., 2011; Wamsley and Fishell, 2017	*Rnd2* Hand and Polleux, 2011 *Rnd2* Hand and Polleux, 2011
Cortical lamination	NPAS4* Spiegel et al., 2014; Wamsley and Fishell, 2017 FEZF2* Tantirigama et al., 2016 SOX5* Lai et al., 2008 CUX1/2* Cubelos et al., 2010 ETV1* Sorensen et al., 2015 OTX1* Arlotta et al., 2005; Lai et al., 2008 ER81* Arlotta et al., 2005 SOX6° Zimmer-Bensch, 2018 LHX1° Zimmer-Bensch, 2018 SATB1° Denaxa et al., 2012; Wamsley and Fishell, 2017 KCC2/SLC12A5° Bortone and Polleux, 2009; Wamsley and Fishell, 2017 MEF2C° Mao et al., 1999; Wamsley and Fishell, 2017 NPAS4° Spiegel et al., 2014; Wamsley and Fishell, 2017	*Bdnf* Wamsley and Fishell, 2017 *Xlr3b, Xlr4b* Cubelos et al., 2010 *Bdnf* Wamsley and Fishell, 2017

*This table provides an overview of transcription factors and their target genes, as well as of other relevant proteins such as KCC2/SLC12A5, being implicated in distinct aspects of corticogenesis. Annotation:*

* excitatory neurons;

*° interneurons.*

On the other hand, the *progressive specification hypothesis* of interneuron development argues that cellular identity is acquired at the post-mitotic stage (Wamsley and Fishell, 2017). For example, POA-originating interneurons express LHX1 at early post-mitotic stages, another member of the LIM-homeodomain protein family, which modulates expression levels of *Epha4* and *Efnb3*, and therefore acts on guided migration of these cells through the ventral telencephalon (Zimmer-Bensch, 2018; Symmank et al., 2019). Interestingly, cortical layer distribution of POA interneurons is also affected by *Lhx1* deletion, which is only expressed in early post-mitotic interneurons, indicating that transcriptional networks at post-mitotic stages also reach out to later stages of migration within the cortical compartment (Symmank and Zimmer-Bensch, 2019).

Another aspect supporting the *progressive specification hypothesis* is that laminar positioning of cortical interneurons is influenced by interactions with the cortical environment (Fishell, 2008; Wamsley and Fishell, 2017), described to rely on activity-mediated mechanisms within microcircuits (Kepecs and Fishell, 2014; Wamsley and Fishell, 2017). Following this idea, interneurons are discussed to express specific genes, such as *Kcc2/Slc12a5*, *Satb1*, or *Mef2c*, being essential to guarantee proper maturation in response to neuronal activation as soon as they begin to settle within the cortex (Mao et al., 1999; Bortone and Polleux, 2009; Denaxa et al., 2012). The *progressive specification hypothesis* is also underlined by *Fezf2* knockout studies, reporting on improper cortical positioning of projection neurons and subsequent abnormal lamination of interneurons, emphasizing that interneuron distribution depends on cortical environment (Valcanis and Tan, 2003; Tantirigama et al., 2016; Zimmer-Bensch, 2018).

Another crucial factor in the transcriptional network of developing interneurons is the zinc-finger protein ZEB2 (Zimmer-Bensch, 2018). Its reported functions support both hypotheses, since ZEB2 is closely linked to fate determination of MGE-interneurons by regulating *Nkx2.1* expression and by promoting MGE-characteristic genes as *cMaf, Mafb*, or *Cxcr7* (McKinsey et al., 2013; Zimmer-Bensch, 2018). In addition, ZEB2 also directly orchestrates migration of MGE-derived cells through *Unc5b* expression (McKinsey et al., 2013). Moreover, post-mitotic loss-of-function mutants of *Zeb2* failed to perform proper MGE-cell migration by switching to striatal fate, instead of migrating toward the cortex (McKinsey et al., 2013).

Similar aspects serving both hypotheses of interneuron fate and maturation determination are described for COUP-TFI and TFII, both required for guiding CGE-derived cells along specific routes during tangential migration, and potentially influencing subclass fate and laminar positioning of MGE-derived cortical interneurons (Kanatani et al., 2008; Lodato et al., 2011b; Zimmer-Bensch, 2018). Moreover, COUP-TFI is suggested to act on IPC division, therefore controlling the balance of MGE- and CGE-derived cells (Lodato et al., 2011b). Another essential transcription factor known to be exclusively expressed in CGE-derived cells is PROX1, which regulates both migration and maturation of these interneurons (Miyoshi et al., 2015).

Alike for cortical interneurons, progenitor specification and progressive specification hypotheses are discussed for excitatory projection neurons (Guzelsoy et al., 2019). There is evidence that transcriptional networks and genetic programs influence their subtype determination and development, comprehensively reported in several studies to which we refer for more details (Bayer and Altman, 1991; Arlotta et al., 2005; Lodato et al., 2011a, 2015; Sorensen et al., 2015; Guzelsoy et al., 2019; Loo et al., 2019; Di Bella et al., 2020; Fan et al., 2020). Briefly, individual projection neuron subclasses are characterized by a specific set of marker genes (Guzelsoy et al., 2019; Table 1), which at least in part seem to drive their development and maturation (Bayer and Altman, 1991; Arlotta et al., 2005; Lai et al., 2008; Watakabe et al., 2012; Guzelsoy et al., 2019; Fan et al., 2020).

The improvement and increased accessibility in platforms enabling single cell transcriptomic profiling has helped to achieve relevant progress in classifying cortical cell-types in rodents and humans (Darmanis et al., 2015; Hodge et al., 2019; Loo et al., 2019; Fan et al., 2020), in part in combination with morphological and electrophysiological studies (Gouwens et al., 2020). Nevertheless, many aspects of how cortical neurons arise from progenitors and which regulatory elements influence the tightly regulated programs of neuronal development, remain elusive. Moreover, it is still unclear to which extent intrinsic programs as well as environmental cues of individual cells contribute to proper corticogenesis, and how they influence each other. In this context, epigenetic mechanisms of transcriptional control call increasing attention, capable of integrating environmental information in the genome during the development of the healthy brain, but also in the etiology of associated diseases such as NDDs.

## Implications of Epigenetic Mechanisms in Neurodevelopmental Diseases

Extended usage of innovative next generation sequencing techniques for the analysis of human samples and cells from different animal models provide evidence for the epigenome holding a critical function in nervous system development and disease manifestation. Epigenomic signatures include histone variants and modifications, alterations in nucleosome positioning, DNA methylation, and non-coding RNAs. In rodents but also in humans, a variety of mutations have been shown to affect proper function of several chromatin regulators being involved in epigenomic reconfigurations, which lead to incorrect development of the brain and the manifestation of NDDs (Table 2). Studies of children with neurodevelopmental defects indicate that DNA methylation and histone modification are crucial for normal brain development (Cristancho and Marsh, 2020). Additionally, correct transcriptional regulation by ATP-dependent chromatin remodeling and regulation through regulatory non-coding RNAs (ncRNAs; e.g., miRNAs and lncRNAs) have been confirmed to play an essential role in neurodevelopmental processes as well (Van Bokhoven, 2011; Jakovcevski and Akbarian, 2012; Rangasamy et al., 2013; Ronan et al., 2013; Torres and Fujimori, 2015). Changes in epigenomic signatures are suggested to be acquired *de novo* on chromatin but are also discussed to be inheritable, passing from one generation to the next, which is reported for *Caenorhabditis elegans* or mice (Morgan et al., 1999; Gaydos et al., 2014; Wei et al., 2014). In humans, different suggestions debate the problem whether the genetic background of patients causes NDDs, or whether environmental stimuli increase the risk of improper brain development (Buiting et al., 2003; Mastrototaro and Sessa, 2018; Ciptasari and van Bokhoven, 2020; Cristancho and Marsh, 2020). In this section, we provide an overview of different epigenetic mechanisms governing neurodevelopmental processes, which are suggested to be associated with NDDs and MCDs, and to be responsive to “environmental insults.”

**TABLE 2:** Overview of epigenetic key players associated to different NDDs as revealed from studies in humans and mice.

Diagnosis	Critical epigenetic regulator/interaction
Angelman syndrome (AS)	*miR-708*°Maranga et al., 2020 MECP2*/°Williams et al., 2010; Mastrototaro and Sessa, 2018; Maranga et al., 2020 SETDB1* Zhu et al., 2020
Anxiety disorder	DNMT1°Hutnick et al., 2009; Noguchi et al., 2016
ATR-X syndrome	H3K9me3* Mastrototaro and Sessa, 2018 MECP2* Bérubé et al., 2005; Nan et al., 2007
Autism spectrum disorder (ASD)	SETD2* Mastrototaro and Sessa, 2018 ANKRD11, HDAC3°Gallagher et al., 2015; Ockeloen et al., 2015; Mastrototaro and Sessa, 2018; Deurloo et al., 2019; Little and Dwyer, 2019 BAF complexes* Mastrototaro and Sessa, 2018 *ST7OT1-3*, ST7OT1-4* Vincent et al., 2002 SETDB1* Cukier et al., 2012; Zhu et al., 2020 MECP2, MDB5/6* Cukier et al., 2012
Down syndrome (DS)	MECP2 Patkee et al., 2020 *miR155*, miR802, miR125b* Siew et al., 2013 NRON°Willingham et al., 2005; Arron et al., 2006
Epilepsy (chronic)	DNMT1/3A* Henshall and Kobow, 2015; Dixit et al., 2018 *miR-17*∼˜92 cluster, miR-21, miR-155, miR-4521, miR-323a-5p, let-7f, miR-31, miR-34a* Dixit et al., 2018
Fragile-X syndrome	*miR-19b*, *miR-92b*, *miR-125b, miR-132, miR-302b*, miR-323-3p, miR-363, miR-367* Siew et al., 2013
ICF syndrome	DNMT3B*/°Miniou et al., 1997; Kondo et al., 2000; Jin et al., 2008
Kabuki syndrome	KMT2D*/°Gabriele et al., 2018 KDM6A* Gabriele et al., 2018
Kleefstra syndrome (KS)	EHMT1, EHMT2* Mastrototaro and Sessa, 2018 EZH2* Mastrototaro and Sessa, 2018 MML3, NR1I3, SMARCB1, MBD5* Mastrototaro and Sessa, 2018
Luscan-Lumish syndrome	SETD2*/°Lumish et al., 2015; Gabriele et al., 2018; van Rij et al., 2018; Rabin et al., 2020
Major depression disorder	SETDB1°Zhu et al., 2020
Prader-Willi syndrome (PW)	SETDB1°Zhu et al., 2020
Rett syndrome (RTT)	MECP2*/°Mastrototaro and Sessa, 2018; Pejhan et al., 2020 *miR30a*, *miR381*, *miR495*°Wu et al., 2010 miR132* Pejhan et al., 2020 *AK081227* and *AK087060*°Petazzi et al., 2013
Schizophrenia (SZ)	DNMT1/3a°Kirkbride et al., 2012; Matrisciano et al., 2013 SETDB1*/°Zhu et al., 2020
Tatton-Brown-Raham syndrome (TBRS)	DNMT3A* Lane et al., 2020; Yokoi et al., 2020
Van-Maldergem syndrome (VMS)	TET1°Cappello et al., 2013
Weaver syndrome (WS)	EZH2* Al-Salem et al., 2013; Lane et al., 2020 NSD1* Al-Salem et al., 2013; Paradowska-Stolarz, 2014; Donovan and Basson, 2017
Williams-Beuren syndrome (WBS)	SETD2* Mastrototaro and Sessa, 2018 LSD1/KDM1A, HDAC2* Mastrototaro and Sessa, 2018; Deurloo et al., 2019
Wolf-Hirschhorn syndrome (WHS)	NSD2°Riazi et al., 2005; Nimura et al., 2009

Annotation: *shown in human, °shown in mice.

### DNA Methyltransferases and DNA Methylation Signatures in Neuronal Development and Neurodevelopmental Diseases

Spatiotemporal control of gene expression through DNA methylation and demethylation is a highly dynamic process during neuronal development, and the most intensively studied epigenetic mechanism.

DNA methylation is catalyzed by DNA methyltransferases (DNMTs), with DNMT1 and DNMT3A being predominantly expressed in the brain (Guo et al., 2014; Jang et al., 2017; Zimmer-Bensch, 2018). DNA methylation at enhancer and promoter sites is associated with transcriptional regulation. Further, DNA methylation can occur in gene bodies and intergenic regions, being involved in repression of repetitive elements, alternative splicing and alternative promoter choice (Smith and Meissner, 2013; Jang et al., 2017). DNA methylation marks affect transcription by diverse mechanisms. For example, methylation of promoter regions can physically impede transcription factor binding, resulting in transcriptional suppression. Moreover, methyl-CpG-binding domain (MBD) proteins interact with methylated DNA, which then recruit other chromatin and nucleosome remodeling factors that drive inactive heterochromatin formation (Cukier et al., 2012). This is in line with the textbook model of repressive DNA methylation in gene promoter regions. Contrasting the well-accepted function of DNA methylation impeding with the binding of proteins without a methyl-CpG-binding domain, several studies proposed that DNA methylation signatures may also serve as binding motifs for discrete transcription factors without a methyl binding domain (Hudson and Buck-Koehntop, 2018). *In silico* studies revealed an increasing pool of transcription factors that are predicted to bind methylated DNA sequences, and emerging scenarios point to new binding motifs for particular transcription factors being created by specific DNA methylation signatures. Likewise, certain transcription factors might even recognize different sequences, dependent on their methylation state (Zhu et al., 2016).

Further, DNA methylation is a dynamic process. In addition to passive DNA demethylation in dividing progenitors, Ten-eleven translocation (TET) family enzyme-dependent mechanisms initiate active DNA demethylation, also being reported for non-dividing cells such as neurons (Kaas et al., 2013; Zimmer-Bensch, 2018). The TET-mediated oxidation of 5-methylcytosine (5mc) to 5-hydroxymethylcytosine (5hmc) and iterative oxidation forms enables active reversion to cytosine by thymine DNA glycosylase (TDG)-mediated base excision repair (Wu and Zhang, 2017), which is also observed in neurons (Kaas et al., 2013).

Dynamic and cell type specific changes in DNA methylation signatures have been reported during cortical development (Lister et al., 2013; Lister and Mukamel, 2015; Mo et al., 2015; Sharma et al., 2016). What remains unclear so far is whether the DNA methylation status drives cellular identity in the first place, or whether specific DNA methylation patterns result from an already programmed lineage restriction. In support of a critical role of DNA methylation in determining cellular identity of NPCs, Santiago and colleagues have found that manipulating essential players of DNA methylation and demethylation in NPCs causes discrete changes in methylation signatures of pluripotency (*Oct4*, *Nanog* and *Tcl1*), and neurogenesis-related genes (*Slit1*, *Wnt3a*, *Dlx2*, *Otx2* and *Rac3*), accompanied by changes in differentiation (Santiago et al., 2020). Other studies observed that DNA methylation catalyzed by DNMTs occurs widely in neuronal progenitors and that DNMT1 suppresses astroglial differentiation from neural stem cells via regulating genes associated with the gliogenic JAK/STAT pathway (Fan et al., 2005; Feng et al., 2005). In the mouse model, a prenatal *Dnmt1*-deficiency in *Nestin*-expressing neural stem cells promoted the differentiation into astrocytes by increasing astrocyte marker glial fibrillary acidic protein (*Gfap*) and attenuating neurogenesis during corticogenesis, which was correlated with more prominent anxiety-like behavior in adult mice (Supplementary Table S1; Hutnick et al., 2009; Noguchi et al., 2016).

On the other hand, DNA demethylation is supposed to drive neurogenesis as well, since TET1 was recently discussed to promote neurogenesis onset in mice (Kim et al., 2016). Increased levels of DNA hydroxymethylation via TET1 in mice was reported to sequentially act at specific cellular transitions and prominently within enhancers of neurogenic genes, shown to be upregulated during neurogenesis (Noack et al., 2019). Using *dCas9-Tet1* manipulations of hydroxymethylation *in vivo*, Noack et al. (2019) and colleagues showed that gain in 5hmC-marks in murine NPCs resulted in a loss of 5mC in the neuronal progeny. Interestingly, this enrichment of 5hmC marks occurred within or at least in close proximity to neurogenic genes, such as *Emx1*, *Prox1*, *Eomes*, *Dll3*, *Tcf4*, *Tubb3*, *Wnt* family members, and *Dchs1.* The cell-adhesion protein DCHS1, for example, is described to play a pivotal role in human and murine corticogenesis, leading to cortical malformations, heterotopia of neuronal populations, and cognitive impairments when being dysregulated in expression, which is described for syndromes such as Van Maldergem’s (Supplementary Table S1; Cappello et al., 2013). Conclusively, Cappello et al. (2013) describe an epigenetically controlled regulation of *Dchs1* through TET1, which is suggested to impair proper corticogenesis and cognitive functions in mice.

Indications for the importance of DNA methylation in the context of NDDs in humans come from patients suffering from the immunodeficiency-centromeric instability-facial anomalies (ICF) syndrome, a rare autosomal recessive disorder known to be caused by biallelic missense mutations in *DNMT3B*, leading also to cognitive and intellectual disability (Figure 1 and Table 2; Miniou et al., 1997; Kondo et al., 2000; Jin et al., 2008). In affected patients, *DNMT3B* mutations cause DNA hypomethylation of genes relevant for neurogenesis, neuronal differentiation and migration (*LHX2*, *ROBO1*, *CXCR4*, *IFRD2*, *DTX4*, *ENC1*, *JARID2*, *SEMA3B*, *ITM2B)*, as well as intercellular signaling (*CXCR4*, *IL1R1*, *IL1R2*, *TNFRSF19*, *CCR7*, *XCL1/2*, *CCR6*, *CCR1*, *TNFSF11*, *IL8)* (Supplementary Table S1; Jin et al., 2008). Another example for the relevance of DNMTs in brain development and NDDs is the Tatton-Brown-Rahman syndrome (TBRS), a rare neurodevelopmental congenital anomaly syndrome that manifests with overgrowth, macrocephaly, and characteristic facial features, sometimes accompanied by autism spectrum disorder (Figure 1 and Table 1; Yokoi et al., 2020). Patients with TBRS are characterized by mutations in *DNMT3A*, leading to different variants of this methyltransferase (Lane et al., 2020). However, underlying connections to the observed cortical anomalies like cerebral overgrowth or metabolite accumulations in the prefrontal cortex in TBRS patients remain largely unclear so far (Tenorio et al., 2020; Yokoi et al., 2020).

**FIGURE 2:**
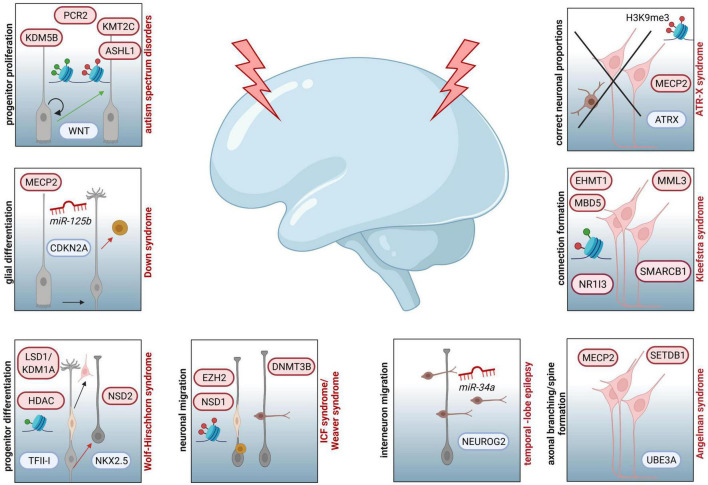
Examples of NDDs involving epigenetic key players and affected processes during corticogenesis assumed to contribute to the respective diseases. Increased progenitor proliferation and decreased neurogenesis due to dysregulated function of WNT can be traced back to aberrant activity of PCR2, KMT2C, KDM5B or ASHL1 in ASD. In individuals affected by Down syndrome, *miR125b* and MECP2 are associated to altered *CDKN2A* expression and glial proliferation. For patients with Wolf-Hirschhorn syndrome neuronal progenitor proliferation is disturbed by aberrant activity of LSD1/KDM1A and HDAC, both targeting the transcription factor TFII-I, as well as dysregulated function of NSD2, which is influencing the expression of *NKX2.5*. In case of ICF syndrome, associated mutations in DNMT3B are resulting in hypomethylated genes essential for neuronal migration (*LHX2, ROBO1, CXCR4, IFRD2, DTX4, ENC1, JARID2, SEMA3B*, and *ITM2*). Additionally, patients with Weaver syndrome are characterized by neuronal migration defects, due to haploinsufficiency of *EZH2*, disturbing PRC2 activity, and mutations in *NSD1*, impairing proper establishment of histone marks. At the level of interneuron migration, different miRNAs are regulating this pivotal step of corticogenesis. For example, downregulation of *miR-34a* is affecting *NEUROG2* expression, which is essential for neuronal migration. Mutations in *UBE3A* and dysregulated expression of *MECP2* and *SETDB1* are linked to susceptibility of Angelman syndrome, which is also characterized by deficits in axonal branching, spine formation and synapse generation. In individuals with Kleefstra syndrome, dysregulated interhemispheric connections are reported as potential result of mutations in *EHMT1*, leading to disturbed interaction with EHMT2 or EZH2 and changes in expression of genes coding for epigenetic regulators, such as *MML3, SMARCB1, NR1I3*, or *MBD5*. Deficits in ATRX-MECP2 interaction, subsequent aberrations in H3K9me3 marks and resulting improper neuronal proportions in different cortical and subcortical areas are depicted for ATR-X syndrome.

In addition to neurogenesis, DNA methylation and DNMT function are also implicated in the regulation of neuronal migration, which is suggested to lead to different NDDs when being disrupted (Figure 1 and Supplementary Table S1; Copp and Harding, 1999; Gleeson, 2001; Pilz et al., 2002; Heavner et al., 2020). Migratory deficits of projection neurons and inhibitory interneurons are suspected to cause numerous neuropsychiatric defects, such as ASD, Tourette syndrome, epileptic seizures, or SZ, which form an own category of neuronal migration disorders (NMDs) (Figures 1, 2 and Supplementary Table S1; Copp and Harding, 1999; Pilz et al., 2002; Guarnieri et al., 2018). A crucial role of DNMT1 in regulating cortical interneuron migration and survival was determined in mice (Pensold et al., 2017; Pensold and Zimmer, 2018). Here, a non-canonical function of transcriptional regulation has been shown through a crosstalk of DNMT1 with histone modifications (Pensold et al., 2017; Symmank et al., 2018). In more detail, it is suggested that DNMT1 promotes the survival of developing interneurons by repressing *Pak6* expression through interaction with EZH2, thereby contributing to the proper establishment of repressive H3K27me3 histone marks (Zimmer-Bensch, 2019b).

In the context of NDD onset, it was shown that manipulating *Dnmt1* expression levels in embryonic cortical interneurons elicited SZ-like phenotypes in offspring (Matrisciano et al., 2013). Further, in patients suffering from SZ, a significant upregulation of *DNMT1* has been found *postmortem* in GABAergic interneurons, shown to cause hypermethylation of *RELN*, which is coding for Reelin and known to be of critical importance for proper corticogenesis (Supplementary Table S1; Kirkbride et al., 2012).

DNA methylation has also been reported to regulate the survival and maturation of cortical projection neurons. As shown by *in vitro* and *in vivo* studies, cortical neuron survival is sustained by proper TET2 function (Mi et al., 2015) and DNMT1 activity in different neuronal subtypes (Fan et al., 2001; Hutnick et al., 2009; Chestnut et al., 2011). Moreover, DNMT1 promotes the post-migratory maturation and refinement of cortical excitatory neurons (Hutnick et al., 2009). For ASD and SZ, recent evidence implicates structural alterations of spiny synapses from glutamatergic projection neurons in affected patients (Dong et al., 2018), which could also rely on developmental defects.

DNA methylation seems further critical in the context of the onset and progression of epilepsy (Henshall and Kobow, 2015; Boison, 2016). Chronic epilepsy represents one of the most prevalent neurological conditions, characterized by different forms of seizures and diverse associated comorbidities (Supplementary Table S1; Henshall and Kobow, 2015; Boison, 2016). Current state of knowledge suggests that aberrant DNA methylation is associated with chronic epileptic seizures, especially in patients with temporal lobe epilepsy (TLE) (Henshall and Kobow, 2015; Pensold et al., 2020). Comprehensive studies on human post-operative material derived from infant TLE patients provide evidence of changes in DNA methylation status and epileptogenesis, using DNA-methylation and RNA-sequencing analyses (Dixit et al., 2018; Jesus-Ribeiro et al., 2021). Here, Dixit and colleagues report on an inverse correlation between promoter methylation and respective gene expression of candidates involved in cellular signaling (*EGFR, PDGFRA, NTRK3, RPS6KA3, PRKAA1*), synaptic transmission (*KCNH8, DLG1*), neuronal development and cell-cell interaction networks (*NEUROD1, NR4A3, ECT2, BCL6, NF-kB2, BRCA1, UNC5B*), which were differentially methylated in TLE patients (Figure 2 and Supplementary Table S1; Dixit et al., 2018). Additionally, the authors suggested that these changes in methylation status rely on significantly upregulated expression of *DNMT3A* and subsequent increase in *de novo* methylation (Table 2; Dixit et al., 2018). Moreover, current suggestions discuss that epileptic seizures by themselves can induce epigenetic modifications and therefore exacerbate the progression of epilepsy during childhood (“methylation hypothesis of epileptogenesis“) (Jesus-Ribeiro et al., 2021). Over the last years, implications for increased activity of DNMTs as well as DNA hypermethylation have been linked to the development of epilepsy in humans but also in rodent models, reviewed in Jesus-Ribeiro et al. (2021).

Besides the correct establishment of DNA methylation or hydroxymethylation, proper read out of de-/methylated sites by methyl-CpG-binding domain proteins are of certain importance. For example, methyl-CpG-binding protein 2 (MECP2), a crucial epigenetic reader (Guo et al., 2014; Mastrototaro and Sessa, 2018), is reported to function as both transcriptional repressor and activator (Amir et al., 1999; Lyst et al., 2013; Della Ragione et al., 2016), and was shown to be highly expressed in the adult brain with increasing protein levels over postnatal development, suggesting a critical role of MECP2 in synapse and circuit maturation during brain development (Mastrototaro and Sessa, 2018). It was found that MECP2 regulates the expression of neuronal development genes such as *GABRB3*, *BDNF*, *DLX5*, *IGFBP3*, as well as genes relevant for cellular migration and adhesion (*PCDHB1* and *PCDH7*) (Kubota et al., 2013; Rangasamy et al., 2013). Congruent with its function as transcriptional repressor and activator, MECP2 deficiency or increased expression can both lead to NDD- and neuropsychiatric outcomes, such as ASD (Table 2; Ramocki et al., 2009). Mutations in X-chromosome-located *MECP2* are associated with Rett syndrome (RTT), a X-linked dominant and severe NDD (Amir et al., 1999; Kubota et al., 2013). Most of RTT-associated missense mutations of *MECP2* have been found in MBDs, implicating an outstanding role in DNA methylation-dependent regulation of gene expression (Kubota et al., 2013; Mastrototaro and Sessa, 2018). Moreover, it has been demonstrated that dysregulated MECP2 activity and resulting epimutations in maternally imprinted *UBE3A* gene are linked to Angelman syndrome (AS), a NDD associated with hippocampal, cerebellar and cortical malfunctions, causing cognitive and language impairments, microcephaly, ataxia and epileptic seizures (Supplementary Table S1; Williams et al., 2010; Mastrototaro and Sessa, 2018; Maranga et al., 2020).

In contrast, less is known about other MBDs and their relevance for NDDs. Recently, *Mbd1 ^–/–^* mice were reported to exhibit several key deficits associated with ASD, including learning difficulties, impaired social interaction, anxiety, and disturbed serotonin activity due to dysregulated *Htr2c* serotonin receptor expression (Allan et al., 2008). Additionally, missense and deletion mutations in *MBD1*, *MBD2*, *MBD3*, and *MBD4* have been reported in children with autism (Cukier et al., 2010). Rather rare mutations of *MBD5* and *MBD6* have been observed in cases of ASD during family pedigree studies, however underlying mechanisms remain unsolved (Cukier et al., 2012).

### Implications of Histone Modifications in Neurodevelopmental Diseases

In addition to DNA methylation, gene transcription is influenced by post-translational modifications (PTMs) of histone tails (Mastrototaro and Sessa, 2018), including acetylation, methylation, phosphorylation, SUMOylation and ADP-ribosylation amongst others (Lovrečić et al., 2013). These PTMs are reversible, being established by antagonistic sets of enzyme complexes that either attach (writers) or remove (erasers) the respective chemical groups. Dependent on the kind of modification and the modified amino acid residue transcription is either promoted or repressed. For example, histone acetylation, being catalyzed by acetyl transferases (HATs), is associated with transcriptional activation, whereas the erasure of acetyl groups by histone deacetylases (HDACs) leads to chromatin condensation (Mastrototaro and Sessa, 2018). In contrast, histone methylation can be either associated with transcriptional repression or activation, depending on the site and the degree of methylation. While H3K4-trimethylation (me3) leads to open chromatin, H3K27me3 causes gene repression (Lachner et al., 2003; Mastrototaro and Sessa, 2018). These particular histone methylations are recognized and bound by particular readers, which then mediate the transcriptional repression or activation by interacting with respective regulatory proteins or complexes.

Histone methylation and acetylation are suggested to be strongly associated with cognitive abilities, like memory formation or learning (Parkel et al., 2013; Peixoto and Abel, 2013; Kim and Kaang, 2017). Moreover, recent studies suggest a critical role of histone modifications in deficits of cognition and intellectual disability, which are often comorbid symptoms in several NDDs (Supplementary Table S1; Parkel et al., 2013; Peixoto and Abel, 2013; Kim and Kaang, 2017).

Intragenic mutations or microdeletions in the gene coding for the histone methyltransferase 1 (EHMT1) that catalyzes H3K9 mono- and dimethylation - markers of facultative heterochromatin- are reported to cause the Kleefstra syndrome (KS), characterized by developmental delay, cognitive and language impairments, and characteristic facial features (Figure 2, Table 2, and Supplementary Table S1; Mastrototaro and Sessa, 2018). Together with EHMT2, EHMT1 can interact with other proteins such as EZH2 to repress gene transcription (Mastrototaro and Sessa, 2018). Additionally, mutations in *EHMT1* have been related to the aberrant expression of *MML3*, *SMARCB1*, *NR1I3*, and *MBD5*, which encode for epigenetic regulators interacting with EHMT1 (Figure 2; Mastrototaro and Sessa, 2018). In the mouse model of Kleefstra syndrome, mice display synaptic dysfunction, potentially explaining cognitive defects in affected humans, which highlight the importance of epigenetic networks for cognitive capacities in different species (Balemans et al., 2013).

Moreover, allele-specific mutations of genes linked to neurodevelopmental processes and coding for histone-modifying proteins such as *ASH1L, KDM5B* and *KMT2C*, have been recently found in patients with ASD. These mutations have been proposed to influence proliferation and the onset of NPC differentiation by interfering with WNT signaling, thereby leading to structural brain anomalies such as micro-/macrocephaly and cortical malformations (Figures 1, 2 and Supplementary Table S1; Krumm et al., 2014; Cederquist et al., 2020; Ciptasari and van Bokhoven, 2020). Interestingly, the reported genes were also described to regulate polycomb-repressor complex 2 (PRC2) binding, which modulates the balance of NPC proliferation and differentiation (Figure 2; Viré et al., 2006; McLaughlin et al., 2019). Alterations in histone methylation states have been found also for Weaver syndrome (WS), a congenital disorder characterized by prenatal or postnatal overgrowth, macrocephaly, cognitive deficits, pachy- and polymicrogyria and dominant neuronal migration defects (Supplementary Table S1; Gibson et al., 2012). In patients with WS, haploinsufficiency of *EZH2* leads to disturbed functionality of PRC2 and diminished mono-, di-, and trimethylation of H3K27, a repressive histone mark (Figure 2; Gibson et al., 2012). Moreover, patients diagnosed with WS display mutations in gene *NSD1*, which encodes for a H3K36-specific methyltransferase (Figure 2 and Table 2; Douglas et al., 2003; Gibson et al., 2012).

Histone methylation in the context of NDDs is also reported for Wolf-Hirschhorn syndrome (WHS), which is characterized by cognitive impairments, intellectual disability, growth delay, and abnormal craniofacial formations (Mastrototaro and Sessa, 2018). Associated with WHS is the H3K36 dimethyltransferase NSD2, demonstrated to act as transcriptional regulator interacting with the transcription factors SALL1, SALL4, and NANOG in embryonic stem cells (Nimura et al., 2009). Moreover, *in vitro* studies in primary murine and human material provided evidence for a NSD2-linked dysregulated expression of *NKX2.5*, a transcription factor that also drives neuronal differentiation (Figure 2 and Supplementary Table S1; Riazi et al., 2005). Interestingly, the ortholog NKX2.1 transcription factor, reported to govern MGE-derived cortical interneuron development, in turn seems to affect the epigenome in mice, since alterations in histone profiles were observed in Nkx2.1^–/–^ animals (Sandberg et al., 2016).

Another example for the implication of H3K methylation in NDDs is the haploinsufficiency of *SETD2* in ASD, the only known enzyme capable to trimethylate H3K36 (Mastrototaro and Sessa, 2018). Increasing numbers of studies report that ASD is often co-existing with Williams-Beuren syndrome (WBS), caused by a duplication of chromosome region 7q11.23 and associated with language impairment, anxiety, ADHD in humans and visual recognition in mice (Supplementary Table S1; Mastrototaro and Sessa, 2018; Deurloo et al., 2019). Suggested to be responsible for WBS is an aberrant expression of *GTF2I*, encoding for transcription factor TFII-I, which is reported to interact with H3K4- and K9-demethylase LSD1/KDM1A and HDAC2, both less active in WBS (Figure 2, Table 2, and Supplementary Table S1). Interestingly, the transcription factor TFII-I is described to be highly expressed in the prenatal and postnatal developing brain and to regulate gene expression in neuronal progenitors (Deurloo et al., 2019), underlining the relevance of histone modification for proper neuronal development.

Apart from histone methylation, alterations in histone acetylation represent a potential risk factor for NDDs. A well-known example is the ankyrin-repeat domain 11 (ANKRD11) protein, which binds and regulates the histone deacetylase 3 (HDAC3) (Mastrototaro and Sessa, 2018). Loss-of-function mutations in *ANKRD11* have been found to lead to developmental delay, language impairments, hyperactivity, and anxiety (Supplementary Table S1; Ockeloen et al., 2015). In humans, *ANKRD11* is expressed in neurons and glial cells and known to modulate ligand-dependent transcriptional activation of *p53*, which encodes for P53. Studies in mice showed that P53 prevents neuronal cell death, being implicated in size regulation of the neocortex (Little and Dwyer, 2019). *In vitro* studies on neural precursors of mice with a point mutation in the HDAC-binding domain of *Ankrd11* showed an upregulation of several genes that encode regulators of cortical development, including SOX6, NOTCH1, NCOR1, NCOR2, MLL5, or SEMA5B, which suggests that ANKRD11 regulates the expression of genes essential for the normal neural development (Gallagher et al., 2015). This hypothesis is supported by *in vivo* analyses of developing murine cortices and behavior experiments observing ASD-like symptoms in adult *Ankrd^11*Yod/+*^* mice (Gallagher et al., 2015).

Alterations in functions of SETDB1, a histone methyltransferase specifically methylating H3K9 in mice and humans being critically involved in transcriptional repression and local heterochromatin formation, have been described for SZ (Chase et al., 2013). *Postmortem* analyses of cortical material from SZ patients indicated an increased expression of H3K9 histonmethyltransferases, including *SETDB1*, as well as higher levels of H3K9me2 compared to healthy control material (Chase et al., 2013). The highly conserved role of SETDB1 in SZ was also confirmed by using *Setdb1* transgenic mouse models, suggesting that it contributes to the pathophysiology of SZ due to dysregulated formation of chromatin contacts associated with SZ risk loci (Ripke et al., 2014; Zhu et al., 2020). Another example of SETDB1 as potential critical factor for NDDs is discussed for major depressive disorder (MDD), which affects a high number of world wide’s population, displays a prominent comorbidity with diverse neuropsychiatric diseases (Table 2 and Supplementary Table S1), and results from dysregulated milestones during brain development (Melartin et al., 2002; Depping et al., 2018; Lima-Ojeda et al., 2018; Schmitgen et al., 2019; Zhu et al., 2020).

In line with this, mice with *Setdb1* deletion in neural progenitors of the forebrain displayed severe impairments of neural development and high risk of postnatal lethality, potentially relying on dramatically reduced numbers of bIPCs due to decreased proliferation and increased apoptosis (Tan et al., 2012). Additionally, SETDB1 is suggested to critically determine brain development at later stages of corticogenesis, since embryonic *Setdb1* knockout brains of mice exhibited elevated astrogenesis at E18.5 (Tan et al., 2012). Moreover, loss of SETDB1 in the mouse model affected the expression of genes relevant for neurogenesis, neural- and non-neural cell differentiation, signal transmission, or neuronal activity (Tan et al., 2012). Together, due to its importance for proper corticogenesis, SETDB1-related defects during brain development represent a risk for NDDs including neuropsychiatric diseases. For other NDDs, as Prader-Willi syndrome (PWS) or Angelman syndrome, SETDB1 was already described to directly contribute to increased susceptibility (Table 2; Cruvinel et al., 2014; Zhu et al., 2020). Both syndromes share the same genomic region on chromosome 15q11–13, with allele-specific loss contributing either to PWS or AS (Supplementary Table S1; Cruvinel et al., 2014). Finally, SETDB1 has been also linked to the etiology of ASD (Cukier et al., 2012). Here, Cukier et al. (2012) report on *Pro1067del*, a non-synonymous allele-specific mutation, which directly targets the catalytic SET-domain of SETDB1 protein. Moreover, they describe a second variation, *Pro529Leu*, which was significantly increased in patients with ASD, compared with healthy family members. Carrying one or both variants contributed to a variety of neuropsychiatric deficits, characteristic for the autistic spectrum (Cukier et al., 2012). Additionally, evidence for an important role of SETDB1 in ASD was underlined by chromosomal microarray analysis of copy number variations (CNV) of ASD-patients and healthy controls, identifying a deletion mutation at chromosome 1q21.3 encompassing the *SETDB1* gene in patients with ASD (Xu et al., 2016). In line with the role of SETDB1 as risk factor for several NDDs in humans, mice lacking KAP1, a crucial binding partner of SETDB1 (Zhu et al., 2020), displayed a significant anxiety phenotype accompanied by learning and memory deficits (Jakobsson et al., 2008).

### ATP-Dependent Chromatin Remodeling Proteins

Together with histone modifications, chromatin dynamics is also regulated by enzymes using ATP-energy (Mastrototaro and Sessa, 2018). Hereby, histone modifications and ATP-dependent chromatin remodeling are tightly interacting to mediate the cooperated fine-tuning of chromatin states.

One pivotal example in the context of NDDs are the so called (SWI/SNF-like) BAF complexes, known to fate cellular lineages of neural stem cells and their subsequent differentiation during brain development (Mastrototaro and Sessa, 2018). Several mutations in genes relevant for nBAF subunits have been implicated in NDDs (Mastrototaro and Sessa, 2018). In mice, loss of the core ATPase *Brg1* results in critical defects during neurogenesis and gliogenesis. The diminished activity of the complete BAF complex leads to reduction in H3K9 acetylation and an increase in H3K27 bi- and trimethylation, shown to cause abnormal cerebral development (Narayanan and Tuoc, 2014; Narayanan et al., 2015). BAF complexes are involved in regulating neuronal differentiation in the embryonic cortex through the modulation of chromatin accessibility, and the binding to specific histone marks and transcription factors, discussed to be associated with learning difficulties, attention deficits (ADHD), and autism, when being dysregulated (Mastrototaro and Sessa, 2018). Further, BAF complex proteins are also highlighted to interact with SOX2, a transcription factor crucial for neural progenitor identity (Mastrototaro and Sessa, 2018; Zimmer-Bensch, 2018), and to influence the transcriptional control of genes regulating neural development and brain size such as *SCN2A*, *DLG2*, *HMGA2* and *SHANK3* (Wang et al., 2017; Mastrototaro and Sessa, 2018).

A prominent example is the ATR-X syndrome, caused by mutations in *ATRX* gene, affecting the SWI/SNF-like ATPase/helicase domain of the encoded ATRX, which impedes the association of the protein with the DNA, H3K9me3 marks, and other proteins (Bérubé et al., 2005; Mastrototaro and Sessa, 2018). These aberrations lead to dysregulated DNA methylation patterns, especially in repetitive elements such as ribosomal DNA repeats (Mastrototaro and Sessa, 2018). Besides, through its ATPase/helicase domain, ATRX also contributes to transcriptional control via the ATRX-DNMT3-DNMT3L domain, which binds histone H3 tails at H3K4me0K9me2/3 (Yamaguchi et al., 2018). During healthy brain development ARTX is proposed to interact with MECP2, providing a mechanistic link for the intellectual disability and cognitive defects seen in ATR-X syndrome patients, for which deficits of MECP2-ATRX interaction are reported (Gibbons and Higgs, 2000; Nan et al., 2007). Further, studies in *Atrx*-null mice showed that ATRX crucially determines corticogenesis in different aspects, since respective loss of function resulted in reduced neuronal density in the cortex and hippocampus, prominently decreased forebrain size, fewer numbers of neurons reaching superficial layers, and increased apoptosis of cortical progenitors upon differentiation (Bérubé et al., 2005). A more recent study investigated a murine ATR-X model using mice lacking *Atrx* exon 2 and reported aberrant dendritic spine formations of cortical *Atrx^Δ*E*2^-*neurons (Yamaguchi et al., 2018). Moreover, *Atrx*-knockout mice displayed memory and cognitive deficits, comparable to intellectual impairments described for ATR-X syndrome patients (Bérubé et al., 2005; Yamaguchi et al., 2018). Similarities in phenotypes and clinical symptoms of mice and humans are suggested to rely on highly similar structures of *Atrx* and *ATRX*, with the SWI/SNF-like ATPase/helicase domain being highly conserved between mice and humans.

All these studies indicate a pivotal role of chromatin-remodeling proteins, such as ATRX, for proper corticogenesis and potential risk factors for severe syndromes (Gibbons and Higgs, 2000; Bérubé et al., 2005; Yamaguchi et al., 2018).

### Regulatory Non-coding RNAs

Within the last years, gene expression control through non-coding RNAs became of emerging significance also in the context of neurodevelopment and NDDs (Mastrototaro and Sessa, 2018; Zimmer-Bensch, 2019a). Non-coding RNAs are defined as regulatory RNAs not encoding for proteins. They can be distinguished in small and long non-coding RNAs (sncRNAs and lncRNAs, respectively), based on their size, but differing also in biogenesis and function. The microRNAs (miRNAs), small-interfering RNAs (siRNAs) and piwi-interacting RNAs (piRNAs) belong to the group of sncRNAs, and mainly act on translation in the cytosol (Mastrototaro and Sessa, 2018). In contrast, the functional spectrum of lncRNAs, RNA species being longer than 200 nucleotides, is enormously manifold, acting on transcription and post-transcriptional events in the nucleus, and further influencing translation, e.g. by functioning as a sponge for or precursors of miRNAs (Qureshi and Mehler, 2012).

Worth to know is the feature that miRNAs are capable of silencing hundreds of different mRNAs, due to incomplete base-pairing, and therefore impeding the translation of various different target genes (Mastrototaro and Sessa, 2018). Also, in the context of NDDs, non-coding RNAs have been discussed to play a pivotal role in mice but also in humans. In the RTT-mouse model, the miRNAs *miR30a*, *miR381*, and *miR495* are significantly overexpressed and described to repress *Bdnf* (Wu et al., 2010). These findings underline an already observed reduction of BDNF levels in RTT-patients in association with human *miR132* and MECP2 (Pejhan et al., 2020). Interestingly, MECP2 promotes the post-transcriptional processing of *miR199a*, which in turn selectively act at the inhibitors of the mechanistic target of rapamycin (mTOR) protein kinase pathway, and is therefore suggested to guarantee proper mTOR signaling during corticogenesis (Tsujimura et al., 2015). Contrarily, genetic deletion of *miR-199a-2* led to reduction of mTOR activity in the murine brain and recapitulated RTT phenotypes in mice, characterized by *Mecp2* mutations and impaired mTOR functionality (Tsujimura et al., 2015), resulting in deficits during fetal corticogenesis and postnatal neuronal function (Park et al., 2018; Ganesan et al., 2019; Tarkowski et al., 2019).

Another critical aspect of MECP2 in connection with miRNAs was recently described in patients with Down Syndrome (DS), a NDD characterized by cortical and cerebellar malformations and cognitive impairments (Patkee et al., 2020). In induced pluripotent stem cells (iPSCs) derived from DS patients, an overexpression of different miRNAs relevant for neuronal development and corticogenesis has been observed (Siew et al., 2013). Here, Siew et al. (2013) described an overexpression of *miR155* and *miR802*, targeting *MECP2*, or *miR125b*, both suggested to influence *CDKN2A* expression and consequently glial proliferation (Xia et al., 2009; Basavaraju and De Lencastre, 2016; Mastrototaro and Sessa, 2018). Interestingly, *miR125b* upregulation also results in suppression of *EPHA4* (Basavaraju and De Lencastre, 2016), which is reported to be crucially involved in proper guidance of murine interneurons during cellular migration (Zimmer et al., 2011). However, whether *miR125b* indeed controls cortical neuron migration during embryogenesis remains unclear so far.

In children diagnosed with TLE, miRNAs have been described to be differentially expressed compared to healthy controls (Dixit et al., 2018). Aberrant expression of the *miR-17*∼*92* cluster, *miR-21*, *miR-155*, *miR-4521*, *miR-323a-5p*, *let-7f*, *miR-31*, and *miR-34a* has been reported to be positively correlated with increased progression and seizure frequency in TLE (Dixit et al., 2018). Interestingly, *miR-34a* was recently shown to regulate *NEUROG2* expression, an essential factor in mammalian neurogenesis, by directly binding to its 5’-UTR (Dixit et al., 2018). In TLE patients, *miR-34a* was significantly downregulated compared with healthy cortical tissue, leading to a less controlled expression of *NEUROG2.* Hence, the authors propose that a disrupted inhibition of neurogenesis and subsequent dysregulation of neuronal migration and differentiation by increased *NEUROG2* expression could be traced back to lower levels of *miR-34a* in patients with TLE (Dixit et al., 2018).

In addition to miRNAs, lncRNAs are debated to play a key role in the onset of NDDs as well as in proper corticogenesis. In this context, only 10% of the described lncRNAs are ubiquitously expressed, whereas the majority can be found in specific cell types or tissues, with about 40% being expressed in the brain (Mastrototaro and Sessa, 2018). Analyzing the lncRNA transcriptome of heterozygous *Mecp2* ±, mice, which are an established animal model for RTT, Petazzi et al. (2013) observed an outstanding upregulation of the two lncRNAs *AK081227* and *AK087060*. *AK081227* is reported to act as cis-regulator of the gene coding for the GABA-receptor subunit *Rho2* (*Gabrr2*), supporting a role of lncRNAs for NDD-associated syndromes, such as RTT (Petazzi et al., 2013). The lncRNA *NRON*, repressing the nuclear factor of activated T cells (NFAT), represents another lncRNA being involved in corticogenesis and neuronal differentiation in human and mice (Ding et al., 2013; Vihma and Timmusk, 2017). In the mouse model, decreased transcription factor activity of NFAT causes DS-like symptoms such as increased social interaction, augmented locomotor activity, decreased muscular strength and decreased anxiety-related behavior, hence assuming a specific role of *NRON* in down syndrome (Willingham et al., 2005; Arron et al., 2006).

Strong body of evidence for an association of lncRNAs with NDDs and MCDs arouse from ASD-related studies (Luo et al., 2019; Safari et al., 2020; Tamizkar et al., 2021). Mutation-screening analyses of patients affected by ASD identified several rare variants of non-coding loci *RAY1/ST7* and *ST7OT1–3*, which were not detectable in healthy control samples (Table 2; Vincent et al., 2002). In addition, Ziats and Rennert (2013) performed microarray analyses of ASD-affected brains *postmortem* and detected over 200 lncRNAs to be differentially expressed compared to healthy brain tissue. Interestingly, the authors could also show that these lncRNAs were enriched for genomic regions containing genes related to neurodevelopment as well as cortical cell migration, and which are suggested to contribute to psychiatric diseases, such as SZ, when being dysregulated. Additionally, Vincent et al. (2002) and colleagues suggest that the improper expression control of these candidates is provoked by the described enrichment of lncRNA variants found in ASD patients, and potentially represent a risk factor for ASD susceptibility (Vincent et al., 2002).

Unquestionably, the functional spectrum of ncRNAs in brain maturation is far from being fully understood. Further investigations in that direction provide an extended opportunity of a better and more comprehensive understanding of NDDs’ etiology.

### Neurodevelopmental Defects Caused by Environmentally Induced Epigenetic Changes

Over the last years, several studies provided evidence for epigenetic modifications being susceptible to environmental stressors, such as malnutrition and mental stress during fetal and neonatal development (Kubota et al., 2015). In addition to stable epigenetic marks, even persistent changes in the epigenetic make-up can be triggered by short-term as well as long-term stressors. In support of this, Matrisciano et al. (2013) could show that prenatal stress of mother mice provoked elevated *Dnmt1* and *Dnmt3a* expression in GABAergic interneurons at embryonic stages, causing schizophrenia-like behavior in the resulting offspring. In mouse models, chronic stress, but also short periods (45 min) of acute stress trigger diverse reconfigurations of repressive histone methylation marks in the hippocampus, which can also be long-lasting (Stankiewicz et al., 2013). On the other hand, epidemiological studies in humans analyzing different cohorts affected by famine demonstrated that children with mothers exposed to malnutrition during their first and second trimester displayed higher rates of mental disorders and SZ (St Clair et al., 2005; Painter et al., 2006; Kirkbride et al., 2012; Stankiewicz et al., 2013). Long-term analyses were conducted including nutritionally compromised women (around the peak of famine close to the time of conception) and their offspring (Susser and Lin, 1992). At approximately 60 years of age, offspring of women affected by famine exhibited less methylation of *IGF2*, which plays a critical role in early development as well as cognitive functions (Kirkbride et al., 2012). It is suggested, that periconceptional maternal famine exposure potentially influences offspring’s DNA methylation in a lasting manner over life-time (Kirkbride et al., 2012). More comprehensive data is already available for mice, showing that different prenatal nutrition (undernutrition, macronutrient deficiency, micronutrient deficiency and overnutrition) individually changes the epigenome of the offspring (Kirkbride et al., 2012). Nevertheless, subsequent studies in humans have linked maternal periconceptional exposure at the famine’s peak with increased risk of distinct neurodevelopmental defects in offspring – neural tube defects, schizoid diagnoses at age 18, and schizophrenia in adulthood (Kirkbride et al., 2012).

Another example is the documented exposure to mental stress within the 1st week of life in neonatal rats, which was shown to change DNA methylation status in the promoter region of the glucocorticoid receptor gene (*Gr*; also known as *NR3C1*) in the brain, resulting in long-term abnormal behavior (Weaver et al., 2004). In addition to fetal and prenatal stress during neurodevelopment, several studies suggest that extrinsic factors, such as drug or alcohol abuse, mental or neuronal stimulation or environmental chemicals alter the epigenetic status and thereby also brain function (Rimland, 1988; Jessberger et al., 2007; Breitling et al., 2011; Gore et al., 2011; Kubota et al., 2015; Dong et al., 2018). Monozygotic twin studies support a correlation of extrinsically triggered alterations in epigenetic profiles and the onset of NDDs, such as ASD, and ASD-like syndromes like RTT or Fragile-X syndrome (Liang et al., 2019). Extended application of high-throughput genomics in combination with functional studies might enable more detailed insights into the role of epigenetic mechanisms in integrating external signals in the context of NDDs. Moreover, twin studies propose the potential of epigenetic signatures as diagnostic markers as well as therapeutic tools for affected patients, but also following generations and family members (Kubota et al., 2015; Mastrototaro and Sessa, 2018).

## Discussion and Concluding Remarks

Corticogenesis is a highly complex and multifaceted process in humans and rodents requiring tight control. Epigenetic mechanisms have emerged as critical regulatory instances for proper orchestration of different milestones in cortical development at the level of progenitor proliferation, neuronal differentiation, cellular migration, laminar organization of neuronal subpopulations or further neuron maturation. Significant progress has been achieved to correlate aberrant epigenetic control seen in NDDs mechanistically to defective neurodevelopmental processes leading to malformations and dysfunctionalities seen in these diseases, which is discussed in this review. What still remains a major challenge is to dissect the role of disturbed epigenetic signatures as being causative for neurodevelopmental defects or rather the consequence. Here, innovative studies that exploit epigenome editing approaches could provide answers (Liu and Jaenisch, 2019).

Further, examples for the implications of changed epigenomic marks in response to environmental insults in NDDs have been presented. Future research needs to dissect in more detail the processes of how environmental insults lead to discrete changes in epigenomic marks and thereby driving disease pathophysiology. Thereby, the complexity in the crosstalk of the different epigenetic mechanisms and their exact implications in discrete neurodevelopmental processes needs to be taken into consideration, to approach potential therapy strategies for NDDs. Moreover, the applicability of discrete epigenetic changes as diagnostic tools might help for early and correct diagnosis of NDDs. Due to comorbidity of symptoms, exact diagnosis is often difficult. An early diagnosis is of great importance, as during embryogenesis and early postnatal life, the developing brain dynamically adapts to external stimuli (Sheikhi and Saboory, 2015), which can be employed to counteract certain impairments. Several mouse studies demonstrated that educational conditions and enriched environment may ameliorate deficits which rely on neurodevelopmental defects (Kubota et al., 2013). For example, in heterozygous *Mecp2* ± female mice, representing a model for RTT-syndrome, housing in larger-sized cages with a variety of objects decreased anxiety behavior in these animals in adulthood (Kondo et al., 2008).

Together with the evidence of epigenetic modifications being dynamic and reversible (Mastrototaro and Sessa, 2018), it seems plausible that epigenetically provoked alterations of brain development can be avoided by prescribing respective inhibitors of these regulatory mechanisms. In fact, several of these inhibitory molecules are already in use for pharmaceutical treatment and many are in the pipeline of drug development (Mastrototaro and Sessa, 2018). For example, HDAC inhibitors trichostatin A (TSA) and 4-phenylbutyrate along with 5-azadeoxycytidine (5-azadC), a methyltransferase inhibitor, are in established use for the treatment of Fragile-X syndrome (Mastrototaro and Sessa, 2018). Another HDAC inhibitor, valproic acid, is applied in patients with epilepsy or RTT to reduce seizure frequency (Mastrototaro and Sessa, 2018). However, in humans, it was generally thought that therapeutics, targeting epigenetic regulators, would be difficult to develop or even to apply due to low rate of specificity and the large influence of epigenetic regulation during developmental processes. The major limitation relies on potential effects on off-target genes, shown for instance for inhibitors of histone methyltransferases and HDACs (Mastrototaro and Sessa, 2018). Here, it is discussed that using these inhibiting molecules as therapeutic approach, this also promotes the activation of oncogenes and potentially increases cancer risk (Mastrototaro and Sessa, 2018). Thus, current research aims to investigate target-specific treatment of NDDs, for which the CRISPR/Cas9 system is suggested to serve as potential approach (Ricci and Colasante, 2021). Recently, it has been exploited to recover the haploinsufficiency of *Scn1a* in mice, known to be essential in the development of different forms of epilepsy (Supplementary Table S1; Staley, 2015; Ricci and Colasante, 2021). Another study used the Cas9-system to specifically recruit multiple DNMT3A catalytic domains to *Sema6a* promoter in post-mitotic and -migratory glutamatergic cortical neurons, leading to rescued impairments of interhemispheric connections, which are described for ASD or SZ (Ricci and Colasante, 2021). Moreover, different studies provide evidence of CRISPR/Cas9 application for potential treatment of Fragile-X syndrome, specifically targeting hypermethylation of *Fmr1* via dCas9-TET activity, leading to almost complete restoration of FMRP protein expression in mice (Ricci and Colasante, 2021).

Although several approaches have been reported on therapeutics of NDDs, preventing respective disorders should be of first priority. Therefore, future research needs to identify molecular signal cascades and their connection to epigenetic modifications in the healthy and diseased brain. This also includes analyses of spatiotemporal dynamics of epigenetic mechanisms and patterns, hopefully elucidating cause, prevention or treatment of NDDs to increase quality of life for affected individuals and families (Lamsal and Zwicker, 2017; Sampaio et al., 2021).

## Author Contributions

Both authors were responsible for the article’s conceptual design, wrote the manuscript, and agreed to be accountable for the content of the work.

## Conflict of Interest

The authors declare that the research was conducted in the absence of any commercial or financial relationships that could be construed as a potential conflict of interest.

## Publisher’s Note

All claims expressed in this article are solely those of the authors and do not necessarily represent those of their affiliated organizations, or those of the publisher, the editors and the reviewers. Any product that may be evaluated in this article, or claim that may be made by its manufacturer, is not guaranteed or endorsed by the publisher.
