# Supplementary material for: The Epigenome in Neurodevelopmental Disorders
Source: Front Neurosci. 2021 Nov 3;15:776809. doi: 10.3389/fnins.2021.776809 (PMC8595945; doi:10.3389/fnins.2021.776809)
Supplement: Supplementary file 1 [file Table_1.docx]

Supplementary Material

# Supplementary Figures and Tables

## Supplementary Table S1

| **Diagnosis** | **Etiology** | **Dysregulated processes** | **Risk factors** | **Clinical Symptoms** |
| --- | --- | --- | --- | --- |
| Angelman syndrome  (AS) | - malformations occipital lobe ^[1]^ - microcephaly ^[1]^ - imbalance neuronal activity neocortex ^[1]^ - defects synaptic vesicle recycling interneurons ^[1]^ | - axonal guidance and branching ^[1]^ - dendritic spine formation ^[1]^ | *- UBE3A* /Ube3a°* ^[1]^  *- SNHG14°* ^[1]^  *- mTORC1°* ^[1]^  *- mTORC2°* ^[1]^  - chromosomal locus 15q11-13* ^[6]^ | - mental retardation ^[1]^  - increased and inappropriate laughter ^[1]^  -ataxia ^[1]^  - absent speech ^[1]^  - seizures ^[1]^  - hyperactivity ^[1]^ |
| Anxiety  disorders | - deficits in GABAergic neuro-transmission in cortical and limbic areas ^[4]^ | - differentiation of neural stem cells into astrocytes ^[2]^ | - *Gfap°* ^[2,10]^ | - panic/ feeling of treat ^[4,5]^  - social phobia ^[4,5]^  - mood impairments ^[4,5]^  - hypersensitivity ^[4,5]^  - depression ^[4]^ |
| ATR-X syndrome | - microcephaly ^[8]^  ^-^ dysregulated synaptic scaling ^[9]^ | - delayed cortico-  genesis ^[8,9]^  - dendritic spine formation ^[9]^ | *- ATRX* ^[3,7-9,11]^  *-* BDNF° ^[9]^ | - mental retardation ^[7-9]^  - facial characteristics ^[7-9]^  - statue abnormalities ^[8]^  - seizures ^[8,9]^  - cardiac defects ^[8]^  - impaired vision/hearing ^[8]^ |
| Autism  spectrum disorders (ASD) | - region-specific changes in cerebral size ^[20]^  - alterations in grey and white matter ^[20]^  - changes in long-range connectivity  - increased cortical thickness ^[20]^  - changes in cortical neuron organization and subtype-specification ^[20]^ | - radial migration ^[20]^  - rate progenitor proliferation vs. neurogenesis ^[12-14]^  - columnar structure cortex ^[20]^ - cytoskeletal remodeling of neuronal cells ^[22]^  - neuronal cell death ^[23]^ | *- FGF8*, *FGF17** ^[20]^  *- mTOR** ^[20]^  *- WNT** ^[12-14]^  - *AUTS2** ^[22]^  *- ADNP** ^[22]^  *- p53, SOX6, NOTCH1, NCOR1/2 MLL5, SEMA5B** ^[23,24]^  - *CNTNAP2, NRXN1, NLGN4, SHANK3** ^[25]^ | - impaired social interaction  and communication ^[20,21]^  - stereotypic behavior and interests ^[20,21]^  - sensory misperception ^[20,21]^  - developmental and/or motor delay ^[22]^ - aggression ^[20,21]^  - hyperactivity ^[20,21]^  - self-injury ^[20,21]^  - anxiety ^[20,21]^  - depression ^[20,21]^ |
| Down syndrome (DS) | - cortical and cerebellar malformations ^[15]^  - decreased volume cerebrum gray matter ^[26]^  - incorrect establishment neuronal number ^[26]^ | - proliferation glia ^[3,17,19]^  - cortical lamination ^[26]^  - neuronal differentiation, especially from aRGCs ^[26]^ | *- CDKN2A** ^[3,17,18]^  - *EPHA4** ^[18]^  - NFAT° ^[27,28]^  - *Arp2/3*^° [26]^ | - facial characteristics ^[15]^  - intellectual disability ^[15]^ |
| Epilepsy  (chronic) | - imbalance between inhibitory and excitatory activity ^[29]^  - focal cortical dysplasia ^[29,37]^  - aberrant synaptogenesis ^[29]^ | - interneuron migration ^[29]^  - formation of neuronal connections ^[29]^ - neuronal process outgrowth ^[29]^ - incorporation of sodium channels ^[29]^ | *- ARX** ^[29]^  - RELN* ^[29]^  *- MTOR** ^[29,37]^  *- SCN1A** ^[29]^  - BDNF* ^[43]^  - NEUROG2* ^[44]^  ^-^ *EGFR, PDGFRA, NTRK3, RPS6KA3, PRKAA1** ^[44]^  - *KCNH8, DLG1** ^[44]^  - *NEUROD1, NR4A3, ECT2, BCL6, NF-kB2, BRCA1, UNC5B** ^[44]^ | - different forms of seizures ^[63]^  - mood and dissociative symptoms ^[30,31]^ - mood disorders ^[30,31]^  - psychotic disorders ^[30,31]^  - self-harm and suicidal behavior ^[30,31]^  - personality disorders ^[30,31]^ |
| Fragile-X syndrome (Martin Bell syndrome) | - impaired synaptic plasticity ^[16]^  - glutamate and GABA receptor signaling ^[16]^ | - synaptic formation ^[46]^  *-* formation dendritic spines and projections ^[26,47]^  *-* aRGC pool extension ^[26]^  - neuronal positioning cortex ^[26]^ | *- FMR1** ^[16,46]^  *- FXR1P** ^[45]^  - CDH2° ^[26]^ | - facial/cranial anomalies ^[16]^  - aggression ^[16]^  - impulsive behavior ^[16]^  - cognitive impairments ^[16]^  - seizures ^[1]^ |
| Immuno-deficiency, centromere instability,  facial ano-malies (ICF) syndrome | - affected neuro-transmission and synapse function ^[32]^ | - neurogenesis, differentiation and neuron migration ^[32]^  - cytokine- and chemokine signaling ^[32]^ | *LHX2*, *ROBO1*, *CXCR4*, *IFRD2*, *DTX4*, *ENC1*, *JARID2*, *SEMA3B*, *ITM2B ** ^[32]^  CXCR4, IL1R1, IL1R2, TNFRSF19, CCR7, XCL1/2, CCR6, CCR1, TNFSF11, IL8 * ^[32]^ | - immune-deficiency ^[32]^  - mental retardation ^[32]^  - intestinal dysfunctions ^[32]^  - psychomotor impairment ^[32]^  - aberrant facial features ^[32]^  - developmental delay ^[32]^ |
| Kabuki syndrome | - microcephaly ^[64]^ | - proliferation of neural stem cells and neural progenitors ^[64]^ | *- KMT2D*/ Kmt2d°* ^[64]^  *- KDM6A** ^[64]^ | - intellectual disability ^[64]^  - facial characteristics ^[64]^ |
| Kleefstra syndrome (KS) | - synaptic dysfunction ^[35]^  - improper synaptic scaling ^[35]^ | - neuro-developmental delay ^[35]^  ^-^ formation of neuronal networks ^[35]^ | *- MML3*, *SMARCB1*, *NR1I3*, *MBD5** ^[3,39]^  - *BDNF** ^[35]^  - *EHMT1** ^[64]^ | - characteristic facial features ^[3,39]^  *^-^* ASD ^[35]^  - cognitive and language deficits ^[3,39]^ |
| Luscan-Lumish syndrome | - macrocephaly ^[65]^  - hypoplasia ^[66]^ | - neurogenesis ^[67]^  - neural differentiation ^[67]^ | *- Nsd1/2°* ^[64]^  - *SETD2** ^[64-67]^ | - overgrowth ^[64,65]^  ^-^ intellectual disability ^[64,65]^  - ASD ^[64,65]^  - developmental delay ^[64]^  - epileptic seizures ^[64]^  - prominent forehead ^[65]^ |
| Major depressive disorder | - changes in gray matter volume ^[48]^  - aberrant cortical gyrification ^[48,49]^ | - cortical folding dynamics during gyrification period ^[49]^ | - KAP1° ^[40]^  - *SIRT1** ^[51]^  - *LHPP* ^[^*^51]^ | - overwhelming sadness and despair ^[48,50]^  - insomnia ^[50]^  - fatigue ^[48]^  - secondary cognitive difficulties ^[48,50]^ |
| Prader-Willi  Syndrome  (PW) | - changes in gray matter volume ^[52]^  - increased cortical thickness ^[52]^ | - onset corticogenesis ^[52]^  - formation cellular connections ^[52]^ | - chromosomal locus 15q11-13* ^[52]^ | - hypotonia ^[52]^  - overeating disorder ^[52]^  - mild to moderate intellectual disability ^[52]^  - variable range of social and behavioral difficulties ^[52]^  - disrupted sexual development ^[52]^ |
| Rett syndrome (RTT) | - microcephaly ^[36]^  ^-^ aberrant neuronal morphology ^[45]^ | - improper stem cell differen-tiation ^[36]^  - improper formation neuronal somata and dendritic spines ^[41]^ | - BDNF° ^[3,41]^  - *MECP2** ^[3,41]^  - mTOR° ^[62]^ | - mental retardation ^[36]^  - speech impairment ^[36]^  - seizures ^[36]^  - ASD ^[36]^  - ataxia ^[36]^  - stereotypic movements ^[36]^ |
| Schizophre-nia (SCZ) | - enlarged cerebral ventricles ^[53]^  - decrease cortical volume ^[53]^  - loss in neuronal processes ^[53]^  - decreased gray matter cortex ^[53]^ | - improper pro-liferation aRGCs ^[26]^  - premature neurogenesis ^[26]^  - decreased formation of dendritic spines ^[53]^  - increased maturation of cortical microglia ^[53]^ | *- DISC1** ^[26]^  - *Cdc42°* ^[26]^  - *Gsk3β°* ^[26]^  - *NRXN1** ^[25]^  - *ZNF804A** ^[25]^  - *RELN*/*/*Reln°* ^[56]^ | - aberrant thoughts ^[57]^ - hallucinations ^[57]^  - delusions ^[57]^  - social impairments ^[57]^  - high risk of developing Alzheimer’s ^[53]^ |
| Tatton-Brown-Raham syndrome (TBRS) | - cerebral overgrowth ^[54]^  ^-^ macrocephaly ^[54]^  ^-^ metabolite accumulations in prefrontal cortex ^[58]^ | - neuro-developmental regression ^[55]^ | *DNMT3A** ^[54,55]^ | - ASD ^[54,55]^  ^-^ facial characteristics ^[54,55]^  ^-^ anxiety ^[55]^  - psychosis ^[55]^  - schizophrenia ^[55]^  - aggressive behavior ^[55]^  - bipolar disorder ^[55]^  ^-^ intellectual disability ^[55]^  ^-^ joint hypermobility ^[55]^  ^-^ afebrile seizures ^[55]^  ^-^ obesity ^[55]^ |
| Van-Maldergem syndrome (VMS) | - periventricular neuronal heterotopia ^[42]^  - simplified cortical gyri ^[42]^  - reduction gray matter ^[42]^ | - neuronal positioning ^[42]^  - formation gyri ^[42]^ | *- FAT4 ** ^[42]^  *- DCHS1*/*  *Dchs1°* ^[42]^ | - intellectual disability ^[42]^  - craniofacial, auditory, renal, skeletal, limb malformations ^[42]^ |
| Weaver syndrome (WS) | - macrocephaly ^[38]^  - pachymicrogyria ^[38]^  - polymicrogyria ^[38]^ | - neuronal migration defects ^[38]^  ^-^ delayed brain development ^[34]^ | *EZH2, NSD1** ^[20,33,34]^ | - prenatal and/or postnatal overgrowth ^[38]^  ^-^ cognitive impairments ^[38]^  ^-^ facial features ^[38]^ |
| Williams-Beuren syndrome (WBS) | - structural changes in amygdala, hippocampus and neocortex ^[59]^  - aberrant columnar orientation ^[59]^ | - dysregulated gene expression in neuronal progenitors ^[60]^  - improper establishment pyramidal-cell and dendritic density ^[59]^ | *- GTF2I** ^[3,60]^ | - language and learning impairments ^[3,59,60]^  - anxiety ^[3,60]^  - ADHD ^[3,60]^  ^-^ hypersociability ^[59]^  ^-^ changes in sleep cycle ^[59]^ |
| Wolf-Hirschhorn syndrome (WHS) | - microcephaly ^[33]^  - cranial asymmetry ^[33]^ | - improperly regulated neuronal differentiation ^[33]^ | *- SALL, SALL4, NANOG, NKX2.5** ^[33,61]^  *^-^ FGFR3** ^[33]^ | - cognitive deficits ^[3]^  - mental retardation ^[3]^  - growth delay ^[3]^  - aberrant craniofacial formations ^[3]^  ^-^ seizures, epilepsy ^[33]^  ^-^ high mortality rate (30%) in first two years of life ^[33]^ |

**Supplementary Table S1: Overview of NDDs and associated impaired processes during corticogenesis, resulting in neuropsychiatric symptoms.** Annotation: * shown in human, ° shown in mice. [1] (Maranga et al., 2020); [2] (Noguchi et al., 2016); [3] (Mastrototaro and Sessa, 2018); [4] (Martin et al., 2009); [5] (Shen et al., 2012); [6] (Buiting et al., 2003); [7] (Bérubé et al., 2005); [8] (Gibbons and Higgs, 2000); [9] (Yamaguchi et al., 2018); [10] (Hutnick et al., 2009); [11] (Nan et al., 2007); [12] (Ciptasari and van Bokhoven, 2020); [13] (Krumm et al., 2014); [14] (Cederquist et al., 2020); [15] (Patkee et al., 2020); [16] (Siew et al., 2013); [17] (Xia et al., 2009); [18] (Basavaraju and De Lencastre, 2016); [19] (Shapshak, 2013); [20] (Donovan and Basson, 2017); [21] (Newschaffer et al., 2007); [22] (Lasser et al., 2018); [23] (Little and Dwyer, 2019); [24] (Gallagher et al., 2015); [25] (Carroll and Owen, 2009); [26] (Arai and Taverna, 2017); [27] (Willingham et al., 2005); [28] (Arron et al., 2006); [29] (Staley, 2015); [30] (Gorton et al., 2018); [31] (Mendez et al., 1993); [32] (Jin et al., 2008); [33] (Paradowska-Stolarz, 2014); [34] (Al-Salem et al., 2013); [35] (Frega et al., 2019); [36] (Amir et al., 1999); [37] (Jesus-Ribeiro et al., 2021); [38] (Gibson et al., 2012); [39] (Balemans et al., 2013); [40] (Zhu et al., 2020); [41] (Pejhan et al., 2020); [42] (Cappello et al., 2013); [43] (Henshall and Kobow, 2015); [44] (Dixit et al., 2018); [45] (Kim et al., 2016); [46] (Razak et al., 2020); [47] (Kubota et al., 2015); [48] (Depping et al., 2018); [49] (Schmitgen et al., 2019); [50] (Deussing and Jakovcevski, 2017); [51] (Cai et al., 2015); [52] (Manning et al., 2018); [53] (Garey, 2010); [54] (Yokoi et al., 2020); [55] (Lane et al., 2020); [56] (Kirkbride et al., 2012); [57] (St Clair et al., 2005); [58] (Tenorio et al., 2020); [59] (Dasilva et al., 2020); [60] (Deurloo et al., 2019); [61] (Nimura et al., 2009); [62] (Tsujimura et al., 2015); [63] (Berg et al., 2010); [64] (Gabriele et al., 2018); [65] (van Rij et al., 2018); [66] (Rabin et al., 2020); [67] (Lumish et al., 2015)
